# Development and Testing of Robust 3D Printed ZnO/Clay Photocatalysts for Sustainable Wastewater Treatment

**DOI:** 10.1021/acsomega.4c09879

**Published:** 2025-04-15

**Authors:** Sardar Ali, Mohannad T Aljarrah, Awni Al-Otoom, Noor Abdelaziz

**Affiliations:** College of Engineering and Technology, University of Doha for Science and Technology, 24449 Doha, Qatar

## Abstract

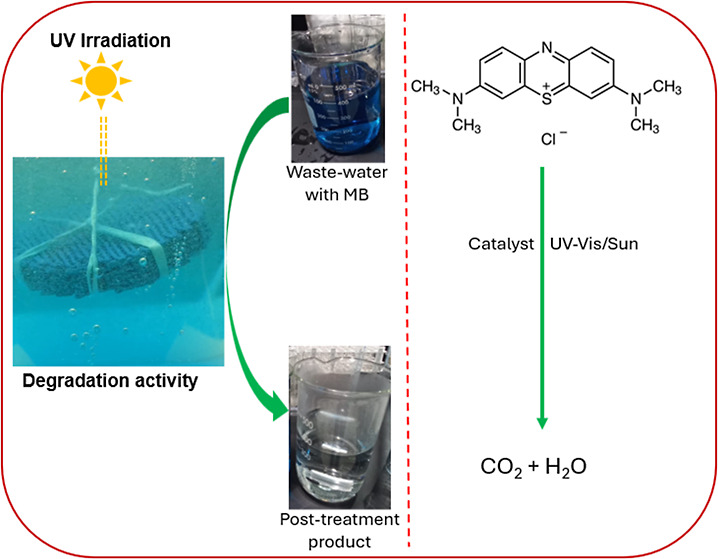

Contamination of water resources with organic pollutants is a serious environmental problem. Degradation of organic pollutants using photocatalytic materials is a promising method of wastewater treatment. In this work, we report on the fabrication of novel 3D printed (3DP) ZnO/Clay materials to be used as efficient photocatalysts for the degradation of methylene blue (MB). Extrusion-based direct ink writing technology was utilized to print the scaffolds. The synthesized scaffolds were thoroughly characterized using the analytical techniques of Brunauer–Emmett–Teller, Fourier transform infrared, X-ray diffraction, scanning electron microscopy, and energy-dispersive X-ray spectroscopy. The photocatalytic activity was studied by measuring the photodegradation of MB under simulated solar radiation. The synergistic effects of adsorption and photodegradation resulted in a 100% removal efficiency of MB within 40 min by the 3DP catalyst. The reported catalysts require minimum time for MB degradation with high pollutants removal efficiency, and therefore, no further treatment is required after usage for the catalytic experiment. The 3DP catalysts also demonstrated excellent stability during the reusability test, where performance of the catalysts was assessed for the degradation of MB using several consecutive cycles without any treatment or regeneration. This work can provide a way for preparing efficient and environmentally friendly photocatalysts.

## 1 Introduction

The demand for water has increased 6-fold during the past century due to rapid industrialization and population growth worldwide.^1^ The manufacturing sector, domestic use, and thermal power generation are the primary drivers of this increase in water consumption.^2^ Water resources have been contaminated by various organic pollutants because of inadequate and inefficient water management, which is a major environmental issue because organic pollutants are highly harmful even at low concentrations.^3^ Organic dyes are of particular concern because of their high toxicity and the wide range of industrial processes that result in wastewater contaminated by dyes, such as those in the food, paper, plastic, leather, textile, and ink industries.^3,4^

Adsorption, reverse osmosis, and ultrafiltration are a few well-known conventional methods for removing organic pollutants from wastewater.^5,6^ Physical treatment techniques are not ecologically friendly since they depend on eliminating organic dyes by transferring it from one phase to another.^7−9^ Because of their complex nature, these organic dyes are rarely biodegradable and tend to persist in the environment.^10^ However, despite their high efficiency, chemical processes like oxidation, chlorination, and ozonation are expensive.^11−13^ Furthermore, chemical methods also use large amounts of hazardous chemicals and result in sludge, which requires further processing and disposal. Recent advancements in wastewater treatment technology have led to sophisticated oxidation methods for the removal of organic pollutants that employ photocatalysts to accelerate degradation without being consumed in the process.^14−16^ The cutting-edge method of photocatalysis is centered on utilizing solar energy as a clean, environmentally friendly, and globally sustainable power source. This technique is based on redox reactions, which produce reactive moieties that can react with organic contaminants and combust them into CO_2_ and water. Advanced oxidation processes (AOPs) have several benefits, including the ability to remove organics rapidly and effectively, the elimination of organic pollutants down to the parts per billion (ppb) level, the absence of hazardous byproducts and sludge, and the elimination of the requirement for toxic chemicals. As a result, AOPs are receiving more attention as a viable substitute for conventional wastewater treatment technologies.^17−20^

Key factors for fabrication of efficient photocatalysts include large surface area, porosity, larger active surface area, and smaller particle size of metals.^21−23^ There have been numerous very active photocatalysts reported in the open literature with excellent performances.^24−33^ The photocatalytic degradation of organic pollutants has been found to be significantly accelerated by catalysts made up of multiple metal oxides, or MOx, including cerium oxide, tin oxide (SnO_2_), nickel oxide, manganese oxide, and silver oxide (AgO).^34−37^ For instance, Tammina and colleagues reported the production of tin oxide nanoparticles and assessed their ability to photodegrade methylene blue (MB) upon exposure to ultraviolet light. They found that SnO_2_-based catalysts with nanoparticles smaller than 3 nm were more effective than those with larger nanoparticles.^38^ However, several limitations hinder the widespread use of MOx systems, including a higher rate of charge carrier recombination, poor stability, and a restricted range of solar spectrum absorption capabilities.^39−41^ Photocatalysts based on TiO_2_ have also been extensively studied. This is because of its better sensing responsiveness, cost-effectiveness, and relatively higher chemical stability. However, the wide band gap of about 3.2 eV severely limits the potential applications of TiO_2_-based materials. Furthermore, the TiO_2_ photocatalyst has a low quantum efficiency.^42^ The effectiveness of TiO_2_-based photocatalysts is significantly influenced by several parameters, including textural characteristics, nanoparticle size, morphology, and the presence of dopants. It has been reported that the addition of carbon-based dopants, specifically graphene oxide, carbon nanotubes, carbon nanofibers, and graphene, significantly enhances photocatalytic activity by accelerating electron transport and lowering charge recombination efficiencies.^43−46^ ZnO-based photocatalysts have been proposed as an alternative to TiO_2_ photocatalysts. This is due to the fact that, despite having the same band gap energy, it has a significantly larger absorption capacity that covers a significant portion of the visible spectrum.^47^ For instance, Fenoll and colleagues conducted a comparative analysis of ZnO and TiO_2_ employed in fungicide photodegradation under comparable reaction conditions and arrived at the conclusion that, when exposed to solar radiation, the ZnO-based photocatalyst was more efficient than the TiO_2_ photocatalyst.^48^ For researchers, developing an efficient photocatalyst for organic contaminant degradation is still a major challenge. Among the main obstacles are the requirement for a photocatalyst recovery procedure, maintaining the catalyst without depletion, and optimizing the process’s total time and cost. One of the main factors limiting the adoption of large-scale photocatalytic processes is the sustainability of the photocatalysts application for wastewater treatment, which is connected to postprocess separation and regeneration, which is frequently difficult and presents economic challenges. Furthermore, valuable metals may be lost because of postprocess separation.^49−52^ As a result, one strategy to deal with these issues is the development of 3D printed (3DP) photocatalysts, which has recently been a prominent area of research recently. This is because 3DP materials can provide several benefits, including porosity, more exposed active areas, and above all ease of postprocess separation and recovery. This method can offer numerous opportunities for creating monolithic photocatalysts with precisely defined porosity and surface area since it uses additive manufacturing to directly fabricate materials with desired properties combined with computer-based design.^53−57^ Although there are several 3D printing techniques, the direct ink writing (DIW) method is likely the most widely used for catalytic materials.^58,59^ For instance, Zhang et al. reported the production of 3DP catalysts using the fused deposition modeling method, which included ZnO nanoparticles supported on ABS/TPU/calcium silicate. The synergistic effects of absorption and photocatalysis contributed to the remarkable efficiency of approximately 97.94% that was recorded when the 3DP catalysts were employed for the photodegradation of RhB dye.^60^ However, 3DP catalysts typically require the use of costly materials and laborious procedures, which raises the cost of the process.

To the best of our knowledge, the integration of zinc nanoparticles with clay to create a 3DP photocatalyst for application in degradation of MB has not been reported in the open literature. The reported research highlights the effectiveness of a robust 3DP photocatalyst, which will serve as an inspiration for novelty in the field. Driven by the aforementioned basis, herein, we report a versatile strategy for successful manufacturing of robust 3DP monoliths consisting of modified clay and decorated with ZnO nanoparticles. Air-dried clay was modified by the addition of alumina to improve its rheological properties and mechanical strength of the paste and to build monolithic scaffolds with tunable porosity. Then, the synthesized zinc powder was added to the paste and printed to the required geometry using extrusion-based technology. The 3DP catalysts demonstrated extraordinary catalytic activities and efficiently degraded the MB dye. Furthermore, 3DP photocatalysts maintain excellent catalytic activity, are highly reusable, and have distinctive characteristics that allow them to be collected directly from water for sustainable wastewater treatment, eliminating the need for a complex separation process.

## 2 Experimental Section

### 2.1 Printing of the 3DP Catalysts

**Figure 1:**
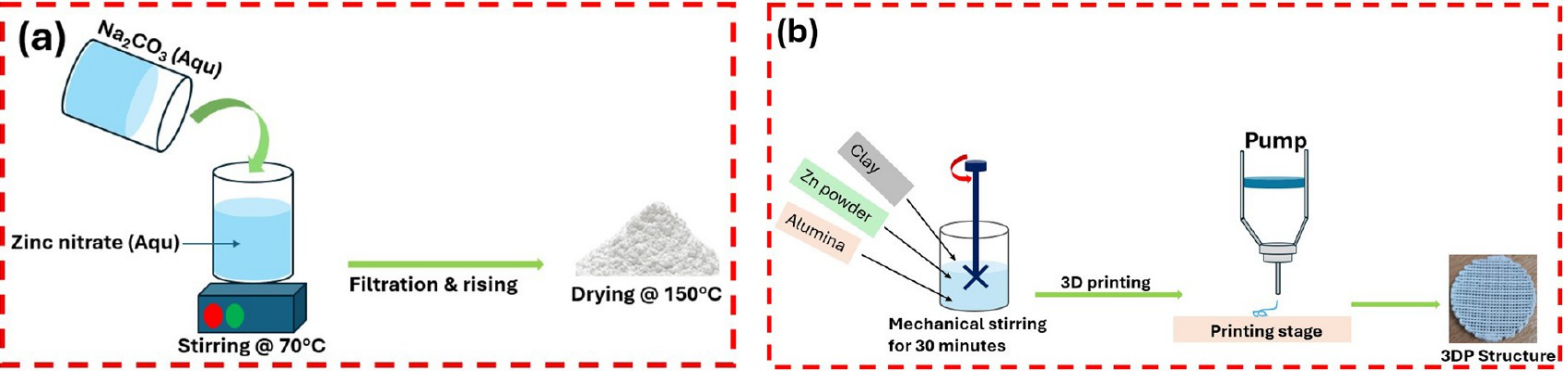
General schematic of 3DP catalysts synthesis: (a) synthesis of ZnO nanoparticles and (b) synthesis of ZnO/Clay 3DP catalysts.

A general schematic of the development of the 3DP catalysts is given in Figure 1. The overall process consisted of two steps with details given below.

#### 2.1.1 Synthesis of ZnO Nanoparticles

The zinc oxide nanoparticles were synthesized using the precipitation method according to the earlier published report.^61^ A standard protocol involved dissolving the required quantity of zinc nitrate hexahydrate [Zn(NO_3_)_2_·6H_2_O, Sigma-Aldrich, >99%] in deionized water in a 500 mL beaker and stirring the liquid with a magnetic stirrer to produce a homogeneous mixture. This was followed by heating the reactant solutions to the required temperature of 70 °C. The aqueous sodium carbonate solution (Na_2_CO_3_, Sigma-Aldrich, >99.5%) was then added dropwise while being stirred vigorously. The resultant precipitate was filtered, cooled to room temperature, and then washed with deionized water several times to remove the excess Na_2_CO_3_. Finally, the precipitates were dried in an oven at 150 °C.

#### 2.1.2 3D Printing of the ZnO/Clay Catalysts

A set of two 3DP catalysts were fabricated. The ZnO-based 3DP catalyst with a composition of eight weight percentage ZnO, one weight percent alumina, and clay was denoted as ZnO/clay. The activity of the ZnO/Al_2_O_3_/clay catalyst was benchmarked with a catalyst denoted as Al_2_O_3_/clay, consisting of 1 wt % Al_2_O_3_. In this procedure, white clay (Jovi, white) was mixed with the required amount of zinc powder, corresponding to 8 wt % on a dry basis. Additionally, the required quantity of alumina (CERALOX, SASOL) was also added in accordance with the weight percentage of unity, and finally, 5 g of water was also added. White clay serves as a matrix and binder for ZnO nanoparticles in this catalytic composition. Alumina was incorporated into the printing mixture to increase the mechanical stability of the scaffolds. To guarantee even mixing, the mixture was vigorously stirred for 1 h using a mechanical stirrer. The mixture was loaded into a syringe attached by a nozzle with a diameter of 1 mm. The printer is equipped with a robotic deposition system to create the required structures with specific geometries. The robotic motion was controlled by the software. A pneumatic pump-powered ink dispenser attached to an air compressor was employed to control the fluid flow rate. The 3DP catalyst consists of a cylinder with dimensions of outer diameter = 25 mm, height = 10 mm, rod size = 1 mm, and space between rods of 0.5 mm. The printing process was carried out under ambient conditions. Finally, the as-printed structure was dried at room temperature for 1 day, dried in an oven at 150 °C, and subsequently calcined at 600 °C for 3 h in air in a furnace at a heating rate of 2 °C min^–1^.

### 2.2 Characterization of the 3DP Catalysts

A detailed characterization of the synthesized 3DP photocatalysts was carried out using various state-of-the-art analytical techniques such as X-ray diffraction (XRD), Fourier transform infrared (FTIR) spectroscopy, X-ray fluorescence (XRF) spectrometry, nitrogen adsorption and desorption [Brunauer–Emmett–Teller (BET)], and scanning electron microscopy energy-dispersive X-ray spectroscopy (SEM–EDX) with elemental analysis. Textural properties of the catalysts were studied using a desktop XRD analysis analyzer (Rigaku MiniFlex). The analysis was performed at room temperature. The analytic technique of N_2_ adsorption and desorption was employed to determine specific surface area, pore size, and pore size distribution using a Micromeritics 2010 surface analyzer. The photocatalysts were also characterized by FTIR using Nicolet iS10 (Thermo Scientific), and all spectra were recorded within the region between 4000 and 500 cm^–1^, at an absolute threshold of 98.614 and a sensitivity of around 50. This analysis starts with degassing the sample by heating it to 150 °C in 50 SCCM of argon gas for 2 h and cooling it down to room temperature to perform the analysis. Specific surface area was calculated using the BET model, whereas pore volume and pore diameters were calculated using the Barrett–Joyner–Halenda model. Morphology of the catalysts was studied by employing SEM (JSM-IT500, JEOL). The XRF analysis was carried out using an Epsilon 1 instrument (Malvern Panalytical). Prior to analysis, the sample was palletized by taking around 7 g of the crushed powder material and mixing 1 g of Elvacite as binding material.

### 2.3 Photocatalytic Activity Experiment

The photocatalytic activity of the synthesized 3DP photocatalysts was investigated by degradation of MB dye as a model of organic pollutants under simulated solar illumination using a 1000 W Tungsten lamp as a source of light. Photodegradation of MB was chosen as a model reaction since it is one of the most prevalent wastewater contaminants produced by various sectors such as pharmaceuticals, paper, and textiles. In this procedure, the scaffold was immersed by hanging it into the solution using a Teflon tap in 400 mL of MB dye aqueous solution, which was prepared by dissolving 10 mg of MB powder in 1 L of deionized water. As presented in Figure S1 in the Supporting Information, the photocatalyst was immersed in the MB solution and stirred for 5 min in the dark under constant stirring to reach adsorption/desorption equilibrium. Directly after that, the sample was exposed to the solar lamp for 1 h. During the experiment, a 3 mL sample was taken at 10 min time intervals and analyzed using a UV–vis spectrophotometer (Agilent, Cary 60). Each of the 3DP catalysts was tested separately to study the composite that has the optimum efficiency toward the photodegradation of MB under the same set of experimental conditions. The recyclability of the best performance of the catalyst was also studied for five consecutive cycles of repeated use. No regeneration was performed between each cycle.

## 3 Results and Discussion

### 3.1 Morphology of the Catalysts

**Figure 2:**
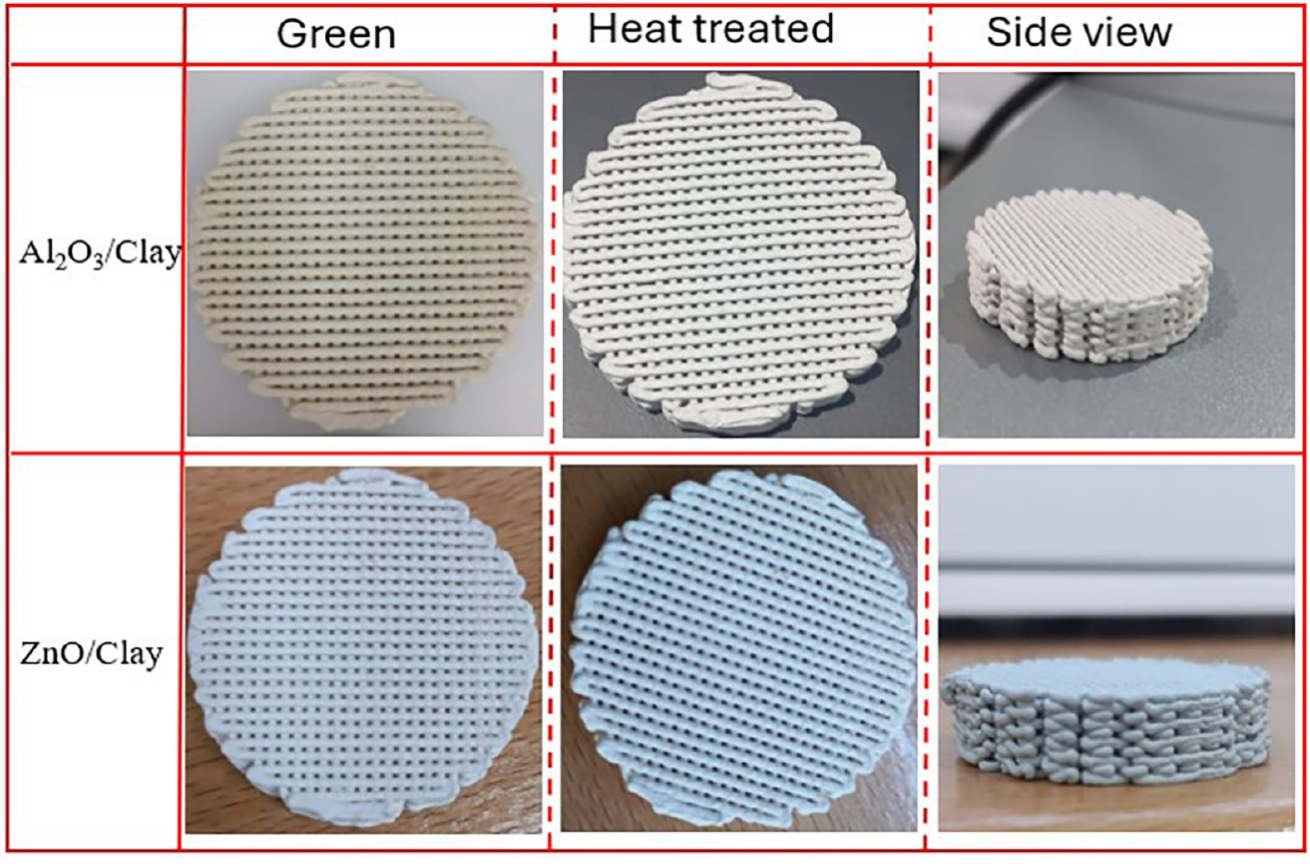
Digital photographs of the 3DP materials taken immediately after printing (green prints), after drying, and after calcination at 600 °C.

Representative digital photos of the catalysts taken following printing, drying, and calcination are shown in Figure 2. The cylindrical 3DP photocatalyst has an outside diameter of 25 mm, a height of 10 mm, a rod thickness of 1.0 mm, and a 1 mm gap between two rods. Following heat treatment, the scaffold’s total mass was approximately 7.8 g. It is crucial to emphasize that following each heat treatment phase, no changes in the dimensions of the scaffolds were noted, demonstrating the structural stability. This contrasts with other reports that showed scaffolds produced using the DIW 3DP technology to have contracted in size.^62−65^ A possible explanation for this might involve the utilization of various printing materials, as opposed to clay in the present case. The loss on ignition of the catalysts was around 30.8%, indicating the presence of high contents of mineral matter.

**Figure 3:**
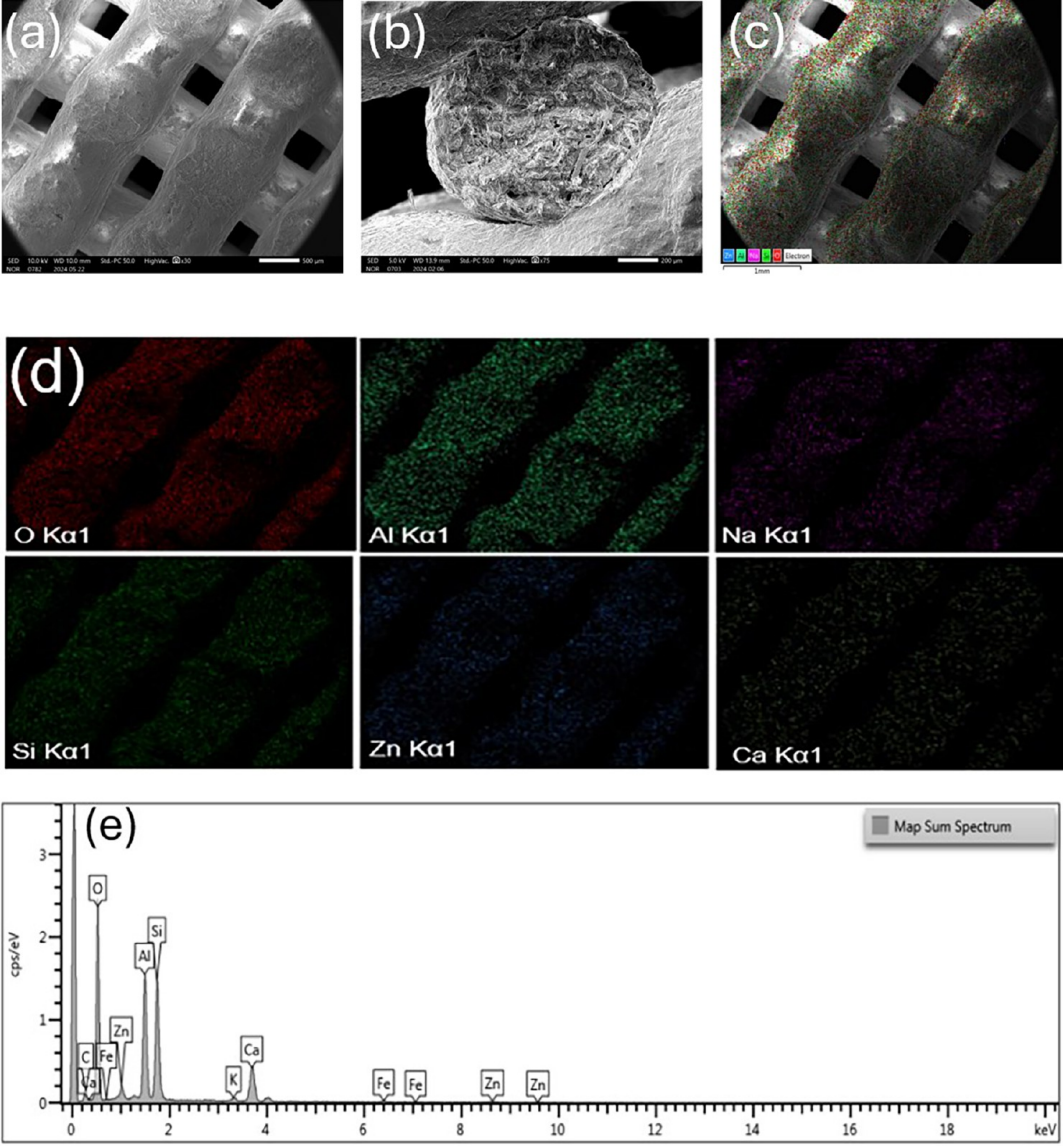
Morphology of the 3DP ZnO/Clay scaffold after heat treatment, (a) SEM image representing the top view, (b) SEM image representing the cross-sectional view, (c) elemental mapping overlay, (d) elemental mapping of individual elements present, and (e) EDX analysis of the ZnO/clay scaffold calcined at 600 °C.

The analytical method of SEM–EDX was employed to examine the morphology of the heat-treated samples, and the results are shown in Figure 3. The findings show that the zinc particles were evenly dispersed throughout the clay network. It is significant to note that no metal particle agglomeration was noticed. Indeed, for effective wastewater treatment, a uniform dispersion of zinc with smaller particles is essential and may result in catalysts’ stable and effective photocatalytic activity. The EDX study results are shown in Figure 3e, where zinc signals are clearly visible in the EDX patterns of the ZnO/clay scaffold, further validating the effective incorporation of zinc.

### 3.2 N_2_ Adsorption and Desorption Analysis

As discussed in an earlier section, one of the crucial factors for efficient photocatalytic activity is surface characteristics of the catalysts. This is because a photocatalyst with a larger surface area will not only alleviate the resistance of mass transfer but also have more exposed surface, resulting in the production of a larger number of reactive radicals and thus making the reaction and degradation more effective. The N_2_ adsorption–desorption technique was utilized to explore surface areas, pores volumes, and pores diameters of the synthesized catalysts. The N_2_ adsorption and desorption isotherms are presented in Figure S2 in the Supporting Information, and analysis data are presented in Table 1. As shown, all catalysts demonstrated type IVa N_2_ physisorption isotherms, indicating the mesoporous structure of the materials with pore diameters > 4 nm.^66^ As can be seen in Table 1, the surface area of the pristine calcined clay was 15.50 m^2^/g, whereas the specific surface areas of the Al_2_O_3_/Clay and ZnO/Clay catalysts were 16.97 and 28.36 m^2^/g, respectively. The pore volumes of the scaffold containing ZnO were also higher than those of the substrate materials. It is worth noting that introduction of the ZnO particles resulted in an increase in surface area and, as is discussed in a later section, enhancing the photocatalytic degradation efficiency for MB.

**Table 1:** N_2_ Adsorption and Desorption Analysis of the 3DP Catalysts

no	sample	SSA_BET_ (m^2^/g)	*V*_pore_ (cm^3^/g)	average pore diameter (Å)
1	clay	15.50	0.06	56.40
2	Al_2_O_3_/clay	16.96	0.08	74.50
3	ZnO/clay	28.36	0.09	110.3

### 3.3 Elemental Composition

The analytical technique of XRF spectrometry was utilized to study the elemental composition of the pristine clay and clay loaded with alumina and zinc as the active ingredient, and results are presented in Table 2. The pristine clay contained oxides of aluminum, silicon, and calcium as major components. The weight percentages of these elements were 25.25%, 33.98%, and 35.43%, respectively, which can be related to the presence of various phases of these elements, affirming the finding from XRD analysis. Indeed, the presence of these elements in the highest proportions agree with the nature of the clay. Amounts of magnesium and iron with percentages of around 1.4% and 2.1%, respectively, were also detected. The sample containing alumina demonstrated similar elemental composition except for an increase in the percentage of alumina understandably to 27.4%. The XRF analysis of the ZnO/clay catalyst demonstrated the presence of ZnO. Interestingly, the percentage of zinc was coherent with the desired theoretical value, indicating successful loading of the active component into the monolith.

**Table 2:** XRF Analysis Results of the Catalysts

#		percentage		
		clay	Al_2_O_3_/clay	ZnO/clay
1	Mg	1.14	1.31	0.85
2	Al	25.35	27.42	25.97
3	Si	33.98	3.45	29.75
4	K	1.90	1.82	1.85
5	Ca	35.43	34.96	31.92
6	Fe	2.1	2.03	1.71
7	Cu	0.002	<0.0015	<0.0015
8	Zn	0.003	0.004	7.95

### 3.4 XRD Analysis

**Figure 4:**
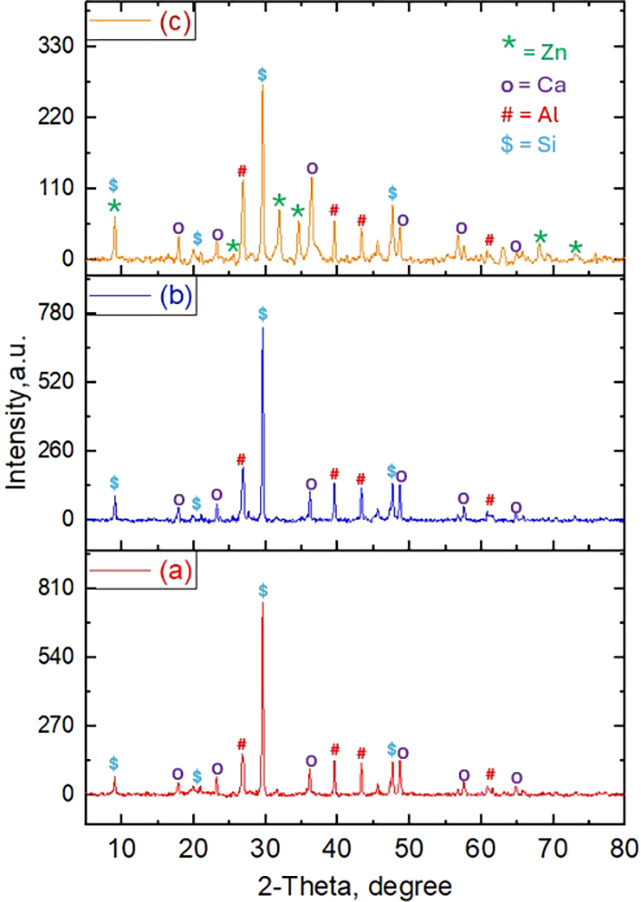
X-ray patterns of fresh and heat-treated scaffolds consisting of (a) clay, (b) Al_2_O_3_/clay, and (c) ZnO/Al_2_O_3_/clay.

The analytical technique of XRD analysis was employed to explore textural properties of the 3DP catalysts in terms of the crystal structure and the presence of various phases. The results are demonstrated in Figure 4. The XRD analysis of the heat-treated clay catalyst revealed the presence of multiple peaks, indicating heterogeneity in the presence of elements, affirming the XRF analysis results. The presence of sharper diffraction peaks indicates a comparatively higher degree of crystallinity that might contribute to stability of the embedded nanoparticles. The diffractions peak appearing at 2θ values of 22.9, 29.6, 46.9, 55.8, and 60.7° can be assigned to various phases of silicon. The distinct diffraction lines corresponding to 2θ values of 28.6, 39.8, 43.7, and 62.7° (marked as #) were assigned to the presence of aluminum oxide.^67,68^ The diffraction peaks appearing at 2θ values of 17.68, 25.43, 37.18, 47.90, 56.52, 58.77, and 66.88° were identified as calcium oxide, which is well in agreement with the earlier literature.^69^ When the XRD patterns of the clay are compared with the spectra of alumina/clay, no deviation from the original pattern is observed, indicating no change in the textural properties of the clay upon incorporation of alumina. It is worth noting that the diffraction line for elements with percentage < 3% was not detected, possibly due to amounts being below detection limits. For the ZnO/Clay 3DP catalyst, additional diffraction peaks were observed that can be attributed to the presence of various phases of Zn. It can also be observed that the peak intensity of the ZnO/Clay catalyst was lower, indicating the homogeneous distribution and smaller particle size of ZnO. The average grain size of zinc oxide was also determined using the Debye–Scherrer equation, which was found to be 31.69 nm. Indeed, the presence of a comparatively sharper peak at 9.6° indicates the formation of several crystal faces of zinc in the 3DP catalyst after calcination. In agreement with the earlier reported literature, additional diffraction peaks for zinc were observed at 2-theta values of 32.67, 35.32, 35.81, 63.66, and 75.69°.^70−72^

### 3.5 FTIR Analysis

**Figure 5:**
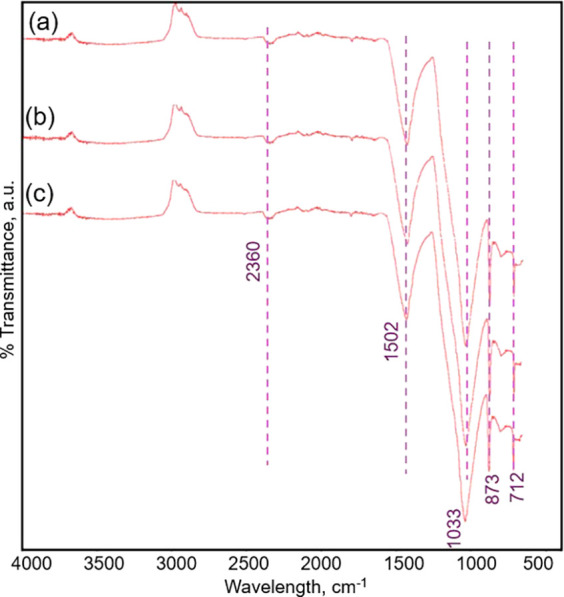
FTIR spectra of the samples, (a) clay, (b) Al_2_O_3_/clay, and (c) ZnO/clay.

FTIR analysis was used to investigate the existence of different functional groups. Figure 5 displays the FTIR spectra of ZnO/clay, alumina/clay, and clay. All three samples had distinctive adsorption peaks at 712, 873, 1033, 1502, and 2360 cm^–1^, according to the FTIR spectra, which were found to be identical. The adsorption peak at 712 cm^–1^, which was ascribed to the stretching vibrations of Si–O–O bonds, is consistent with earlier reports.^73,74^ The characteristic peak at 873 cm^–1^ was also attributed to the OH moiety, which may be the method of deformation of Al–OH–Al and/or Al–Al–OH. Furthermore, the adsorption peak that appeared at 1033 cm^–1^ can be attributed to the typical metal oxide (M–O) stretching bands mostly associated with metal oxide (M = Al, Si, Zn, Ca, etc.) stretching vibrations. The presence of H–O–H bond stretching has been identified as the source of the adsorption peaks that occurred at about 1502 cm^–1^, indicating a possibility of water hydration in all samples. The relatively small adsorption peak at approximately 2360 cm^–1^ originated from stretching of the C=C conjugated bond.^75^

### 3.6 Photocatalytic Activity

#### 3.6.1 Evaluation of Photodegradation for MB Dye

**Figure 6:**
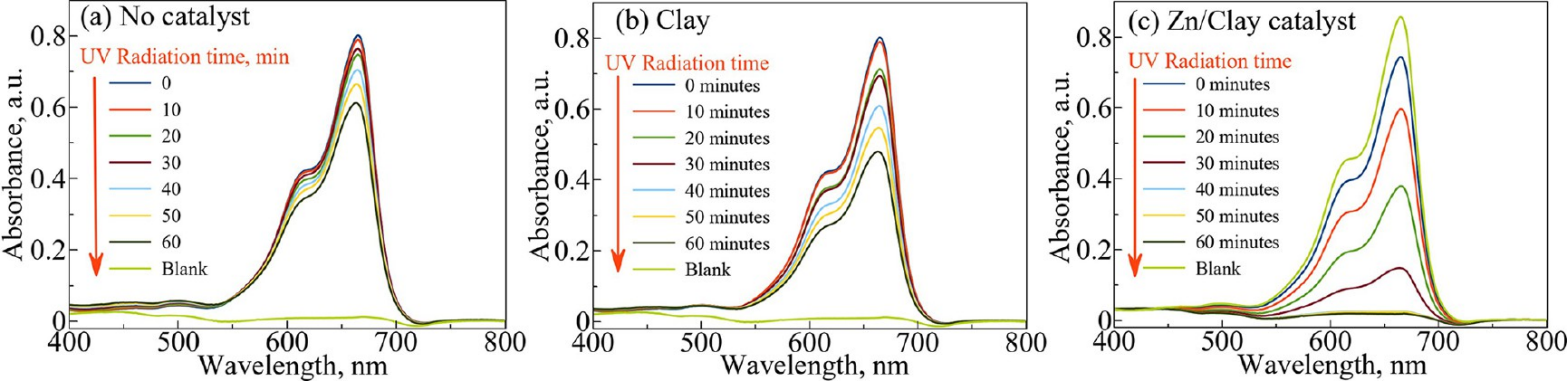
UV–vis spectra of MB dye with (a) no catalyst and (b) Al_2_O_3_/clay and (c) ZnO/clay 3DP catalysts.

**Figure 7:**
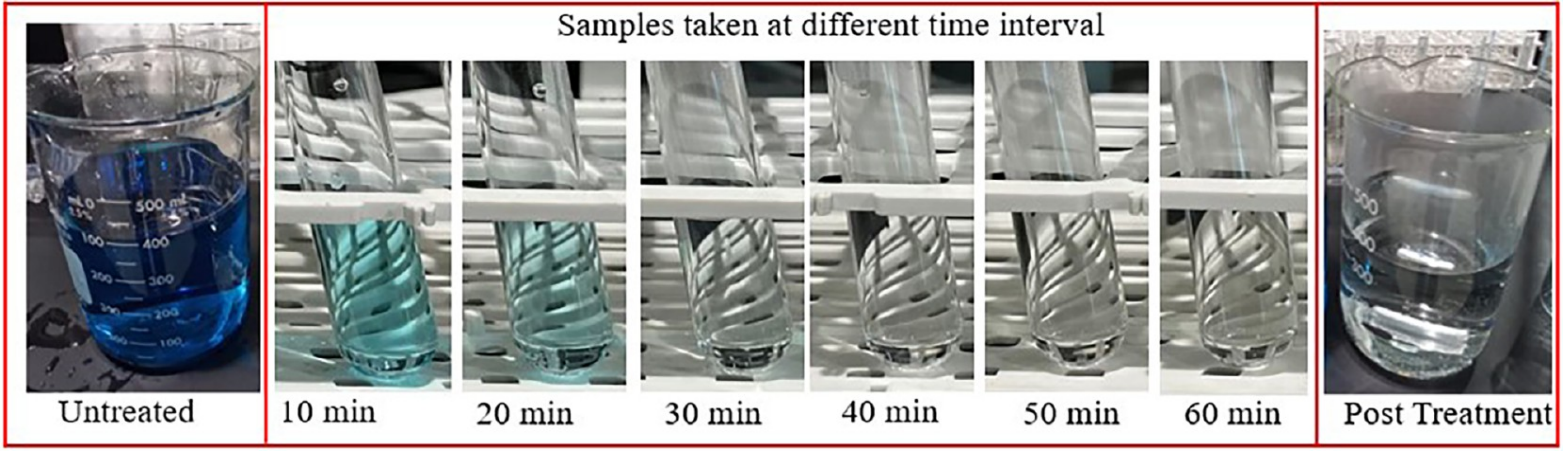
Representative images of the MB dye solution taken before treatment and at different time intervals during photocatalytic activity and post-treatment.

**Figure 8:**
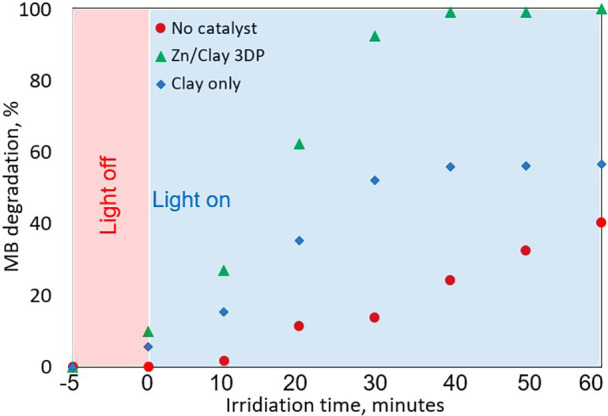
Photodegradation efficiency and concentration changes of MB in the presence and absence of the ZnO/Clay 3DP catalyst.

As previously stated, the photocatalytic activity of the samples was evaluated by the photodegradation of MB dye as a model of organic pollutants under solar illumination by using tungsten light. The photocatalytic activity for all samples was tested under similar experimental conditions. The absorbance changes during the photodegradation processes of MB at different times were measured using a UV–vis spectrophotometer at a λ_max_ of 665 nm for MB dye. As can be seen in Figure 6a, in the absence of the photocatalyst, a very slight decrease in absorbance of MB dye at λ_max_ = 665 nm was observed, and only around 40% photodegradation of MB was achieved within 60 min irradiation. As shown in Figure 6b, after 60 min of exposure to simulated solar light, the clay scaffold was able to decompose about 53% of MB only. A possible rationale for this behavior could be the lack of active sites capable of catalyzing the adsorbed MB. The adsorption of MB on the active sites is likely the dominant phenomenon in its absence; as the reaction time proceeds, the adsorption sites are occupied, preventing further activity for MB decomposition. The employment of the 3DP ZnO/Clay catalyst resulted in a remarkable increase in its photocatalytic activity, as evidenced by the UV–vis spectra of MB dye in Figure 6c. A higher rate of degradation is achieved, reaching an efficiency of 100% removal of the dye. The change in color of the solution taken at different time intervals during the MB degradation experiment over the ZnO catalyst is shown in Figure 7. It is evident that solution color changes from dark blue to light blue and finally to colorless in approximately 30 min of irradiation. This result confirms the role of the presence of the active ingredient and 3DP reactor in enhancing the photocatalytic activity. In the presence of the ZnO/Clay photocatalyst, accelerated photodegradation of MB dye was observed and a photodegradation yield of 100% was obtained for the same irradiation time (Figure 8). The high catalytic efficiency of the ZnO catalyst can be attributed to the larger specific surface area and smaller and uniformly distributed ZnO particles over the substrate, thus providing more exposed active sites for the photocatalytic activity. A similar conclusion has been reported by Liu and co-workers, where increased surface area resulted in catalysts with comparatively high catalytic activity for the MB degradation process.^56^ Pure clay materials have also been shown to be capable of adsorbing MB from aqueous environments. On the other hand, pure clay has very little photocatalytic activity. The synergistic effects of adsorption and degradation, which promote the removal or degradation of MB, are therefore another factor contributing to the high catalytic activity of the ZnO/clay catalyst.

#### 3.6.2 Repeatability Tests and Batch-to-Batch Consistency of the Catalysts

**Figure 9:**
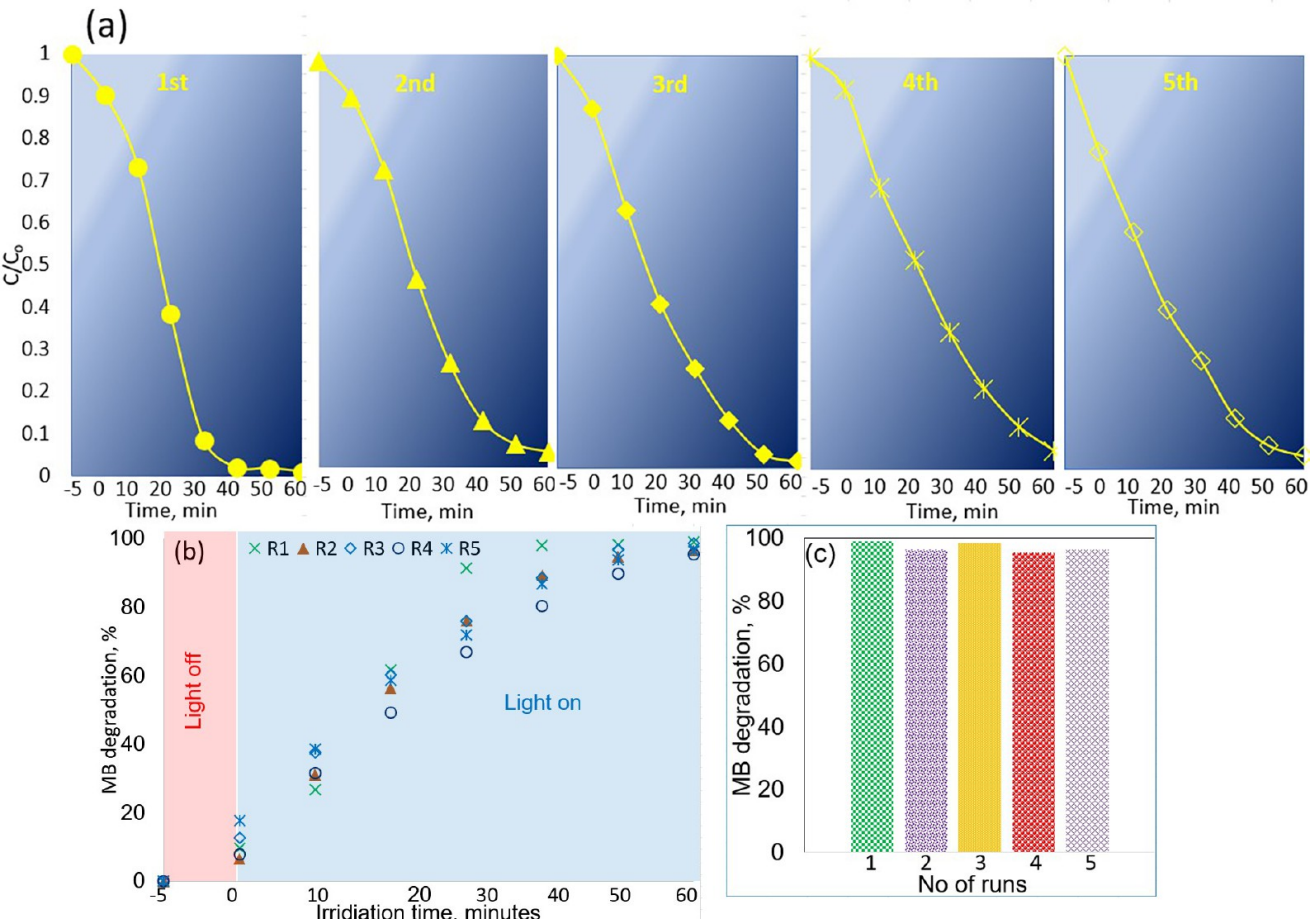
Activity results of the catalyst during five consecutive cycles of photodegradation of MB, (a) comparison of degradation efficiency, (b) comparison of percentage removal of MB, and (c) comparison of photocatalytic efficiency after 40 min of irradiation.

Water treatment applications usually involve the need to treat large amounts of water. For practical and economic reasons, the ability to recycle the photocatalyst with a minimum efficiency loss is very important. In this research, the photocatalytic activity of the catalysts was also studied for several consecutive cycles. Contrary to earlier reported studies, no regeneration was performed in between the cycles.^76−79^ After even five consecutive cycles, no loss in the degradation efficiency was recorded. This is very likely due to the stability of ZnO particles in the 3D structure. As shown in Figure 9, the 3DP ZnO/clay catalyst retained its removal efficiency for five consecutive cycles. As can be seen, the 3DP photocatalyst retained an activity >97% after five consecutive cycles of use. The average photodegradation efficiency with average deviation was 98.12 ± 1.26. In fact, recyclability of the photocatalysts can also be calculated by studying the structural changes during the degradation process. It is important also to highlight that no fracturing was observed after reuse, confirming that the catalyst structure was intact.

**Figure 10:**
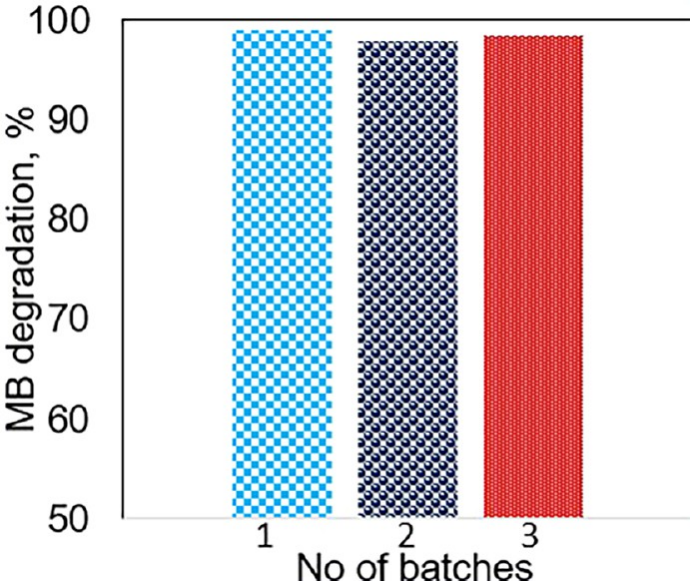
Activity results for three batches of catalysts during MB photodegradation under similar reaction conditions.

Batch-to-batch consistency is one additional fundamental component of catalyst development that refers to the reproducibility of reactions performed in several batches of the same catalytic system under identical conditions. It is important to emphasize that achieving batch consistency is another vital aspect of the catalytic system that is critical not only for scientific research but also for industrial application. Inconsistent batch-to-batch reactions may cause significant fluctuation in catalytic performance, leading to lower product quality and higher costs. For the purpose of this research, three batches of catalysts were produced and tested for MB degradation under similar conditions. The results are shown in Figure 10, and as can be observed, the catalytic performance results of the three batches were similar, demonstrating the possibility of developing reliable and consistent catalysts across several batches.

#### 3.6.3 Postulated Photocatalytic Mechanism

**Figure 11:**
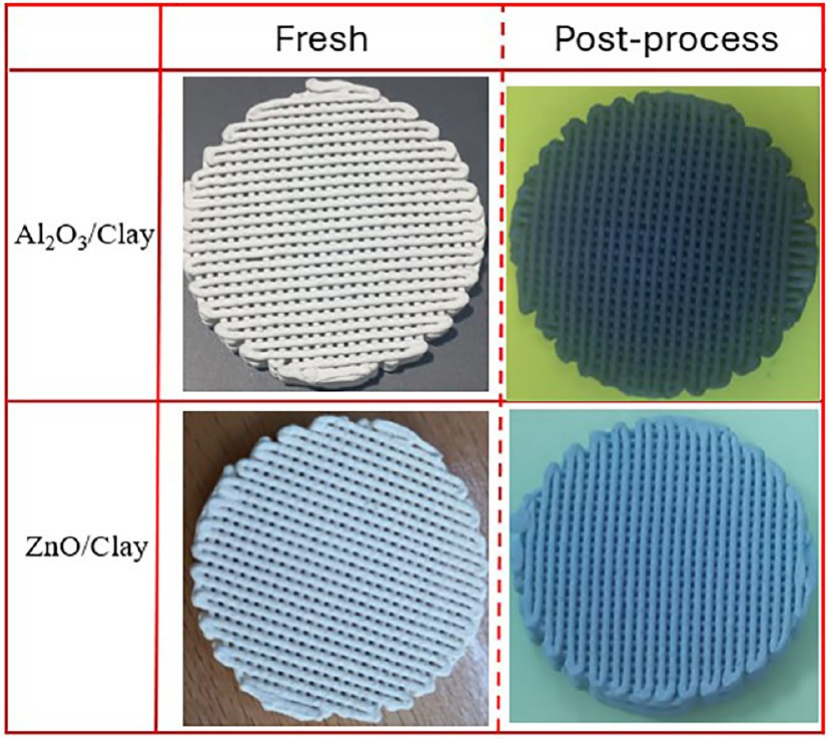
Representative digital photos of the catalysts before and after the MB degradation experiment.

It is essential to define the photocatalytic mechanism of organic pollutants as this understanding can be utilized to optimize the reaction variables to achieve excellent photocatalytic efficiency. A systematic approach was therefore employed to better understand the mechanism of MB photocatalytic degradation. Digital photographs of the used clay and ZnO/clay 3DP samples are shown in Figure 11. As can be observed, the ZnO/clay scaffold turned slightly bluish after being employed for the degradation process, whereas the color of the clay scaffold changed dramatically and turned dark blue. These results imply that on the clay, MB is mostly removed by adsorption, while the ZnO/Clay catalyst is a dynamic process that most likely combines adsorption and photocatalytic degradation of MB.

**Figure 12:**
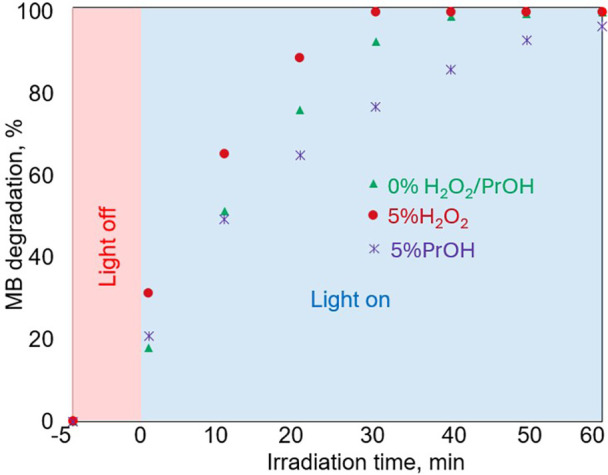
Effects of the presence of H_2_O_2_ on the photocatalytic activity of the ZnO/clay catalyst.

It is generally established that the various reactive species such as ^•^OH (hydroxyl radical), surface cations, and/or holes (h^+^) and ^•^O_2_^–^ (superoxide radical) are responsible for the photocatalytic oxidation of dyes into CO_2_ and H_2_O. However, comprehending the photodegradation process involves determining the active moieties responsible for the degradation process. Therefore, two sets of investigations that include the addition of a sacrificial agent and a scavenging agent were carried out to determine the main active moieties involved in the photocatalytic degradation. In one set of tests, the effects of H_2_O_2_ were examined, while the scavenging effects of 2-propanol in the reactant mixture were examined in the second set of experiments. The findings are displayed in Figure 12. As can be seen, without the addition of H_2_O_2_, the photocatalytic efficiency after 30 min of time was 96.2%, which increased to 100% degradation after 30 min when H_2_O_2_ was added into the reaction mixture. It has been reported that adding H_2_O_2_ to the MB solution acts as a sacrificial agent, enhancing the probability of excited electrons being adsorbed and/or captured on metal active sites, increasing the number of holes on the metal oxide surface, inhibiting pair recombination, and thus improving catalytic activity. Moreover, it has also been reported that addition of H_2_O_2_ results in improvement of degradation activity due to the formation of the oxidant.^80,81^ Additionally, the H_2_O_2_ molecules can also produce OH^–^ radicals in the presence of irradiating photons. These findings clearly indicate that the main active species during the photocatalytic activity are ^•^O_2_^–^, ^•^OH, and h^+^, having a greater impact on the over efficiency of the degradation process. Figure 14 illustrates how the catalytic activity significantly decreased when 5% PrOH was introduced to the reactant mixture; after 30 min, only around 75% degradation of MB was attained. This is because PrOH functions as a radical scavenger and is known to lower the hydroxyl radical (^•^OH) concentration.^82^ This evidence indicates further that in the current scenario, hydroxyl radical moieties may have played an integral role in the photodegradation of MB.

**Figure 13:**
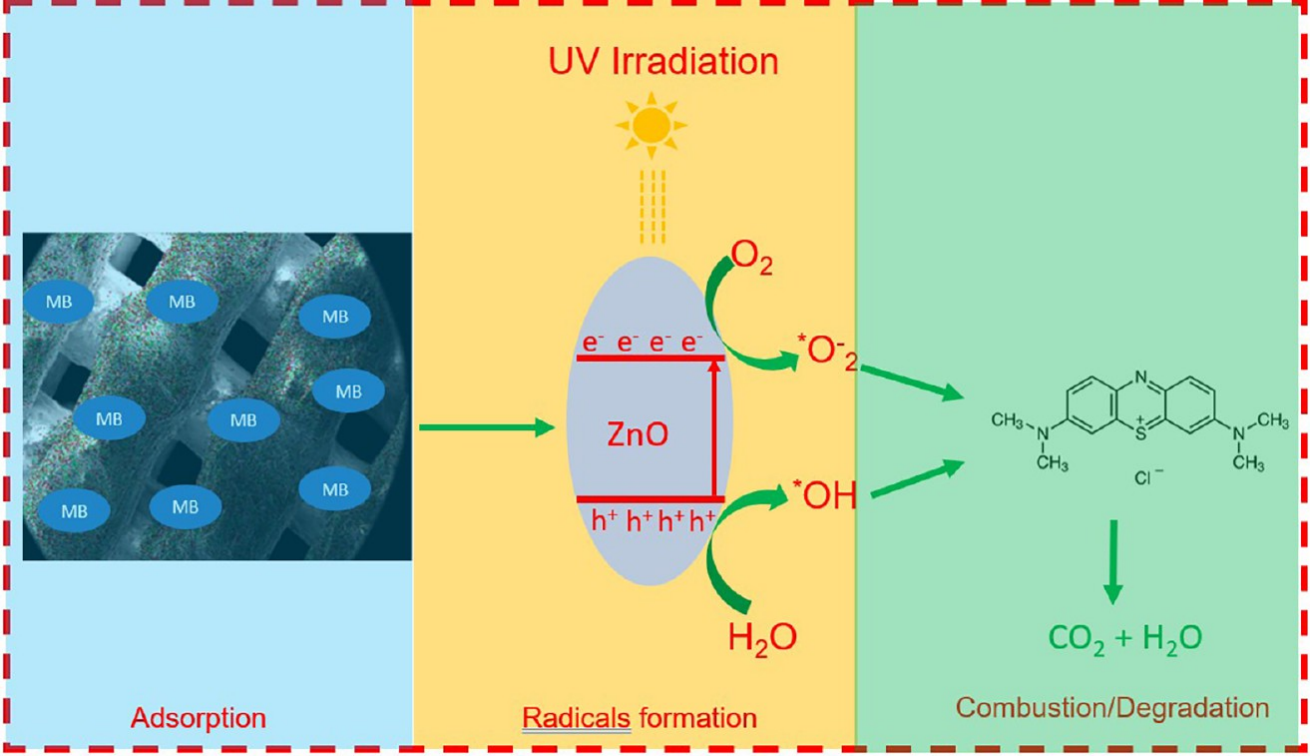
Proposed mechanism of MB degradation by the ZnO/clay 3DP catalyst involving adsorption and photocatalysis.

Based on all of these findings, it can be postulated that the MB degradation over the ZnO/clay catalyst is due to the electrostatic forces and steric effects between the ZnO-coated clay materials and MB (presented in Figure 13). Various functional groups present on the clay substrate presumably tend to stimulate electrostatic interactions with the MB moieties, resulting in excellent adsorption. Previous reports have evidenced enhanced catalytic activity due to stronger adsorption capacities of the photocatalysts. For example, Jin and co-workers reported an increase in photodegradation activity with an increase in adsorption capacity of the catalysts.^83^ After adsorption, the degradation of the MB molecules is mainly driven by the presence of uniformly distributed ZnO nanoparticles upon exposure to light irradiation, which results in release of electrons that in turns react with nearby oxygen molecules (O_2_) to generate O_2_^–^, known as the superoxide anion. The cations present on the surface then leave behind *OH radicals after receiving electrons either from the surrounding moisture and/or from the water present in the reaction medium. The formed O_2_^–^ and *OH radicals are extremely reactive, and their redox power decomposes the MB dye into water and CO_2_ and thus cleans up the wastewater.

#### 3.6.4 Characterization of the Used Catalyst

**Figure 14:**
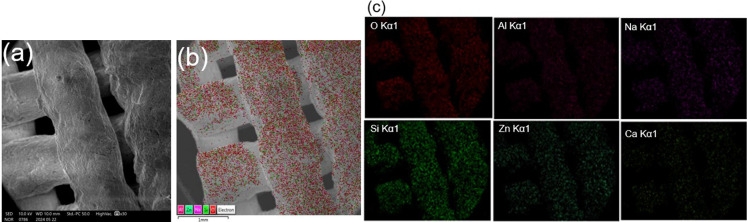
SEM–EDX analysis results of the postrun/used ZnO/clay 3DP catalysts: (a) SEM image, (b) EDX analysis, and (c) high-resolution elemental mapping.

**Figure 15:**
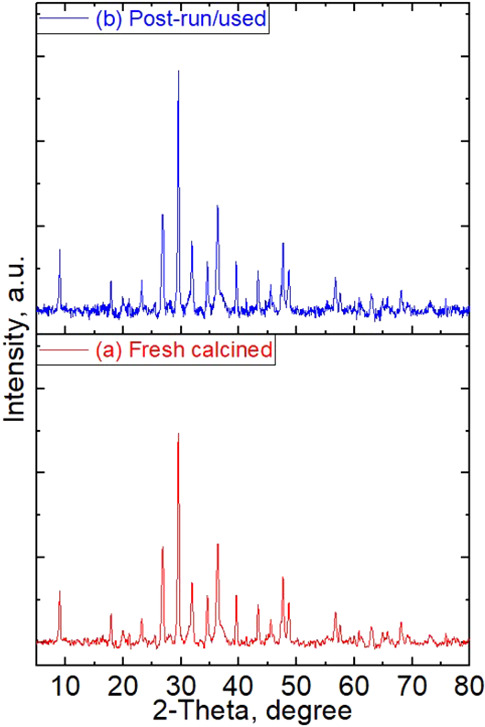
XRD analysis results of the fresh calcined and postrun/used ZnO/Clay 3DP catalyst.

The postprocess catalyst was further characterized by SEM–EDX and XRD analysis to reveal the microstructural characteristics of the catalysts to explain the potential factors that influence reusability of the ZnO/clay catalyst after employment for various conditions. According to the SEM images displayed in Figure 14, the morphology of the ZnO catalyst did not change before and after the MB degradation experiments. Moreover, the EDX elemental mapping results revealed uniform elemental distribution identical to that of the fresh catalyst. XRD patterns of the used sample, in comparison with those of the fresh calcined samples, are shown in Figure 15. The XRD analysis affirmed the findings from SEM–EDX analysis, where no changes in the crystal structure and/or crystallite size were observed. Indeed, it can be concluded from these analysis results that the photocatalytic degradation does not modify and/or alter the structure of the catalyst. This is one of the unique features of the developed 3DP structure with wider applicability in a variety of chemical processes.

## 4 Conclusions

We report on the development and testing of novel clay-based ZnO 3DP catalysts for application in photocatalytic degradation of organic pollutants using MB as a model reaction. The photocatalysts were printed by using extrusion-based technology. The catalyst has a cylindrical shape with an outer diameter of 25 mm, a height of 10 mm, a rod size of 1 mm, and a spacing of 0.5 mm. The as-printed green structure was stabilized by drying it at room temperature for 24 h followed by drying in an oven for 2 h at 150°C and finally calcining it at a temperature of 600°C with a heating and cooling ramp of 1°C/min and a dwell time of 3 h. The structure, surface morphology, and chemical composition of the developed catalyst were studied using XRD, SEM, and EDX analysis techniques. The photodegradation of MB was measured using a UV–vis spectrophotometer. Photodegradation of MB as a model material of organic pollutants showed the enhanced activity of the 3DP catalysts as compared to that in the open literature. Around 100% degradation of MB was achieved in 40 min. Recycling experiments showed good stability, photocatalytic activity, and reusability of the 3DP catalysts under repeated use. The catalyst retained photoactivity >98% after 5 consecutive cycles. Analysis of the used sample did not indicate any change in properties.

## Supporting Information Available

Demonstration of the experimental setup for photodegradation experiments, method of calculating degradation rate of MB, and N_2_ adsorption and desorption curves of the photocatalysts (PDF)
