# Supplementary material for: Development and Testing of Robust 3D Printed ZnO/Clay Photocatalysts for Sustainable Wastewater Treatment
Source: ACS Omega. 2025 Apr 15;10(16):16156–68. doi: 10.1021/acsomega.4c09879 (PMC12044459; doi:10.1021/acsomega.4c09879)
Supplement: Supplementary file 1 — ao4c09879_si_001.pdf [file ao4c09879_si_001.pdf]

Supporting Information

Development and Testing of Robust 3D Printed ZnO/Clay Photocatalysts for Sustainable Waste-water Treatment

Sardar Ali*, Mohannad MT Aljarrah, Awni Al-Otoom and Noor Abdelaziz

College of Engineering and Technology, University of Doha for Science and Technology, 24449 Doha, Qatar

Supplementary Information

1. Experimental set up for photodegradation experiments

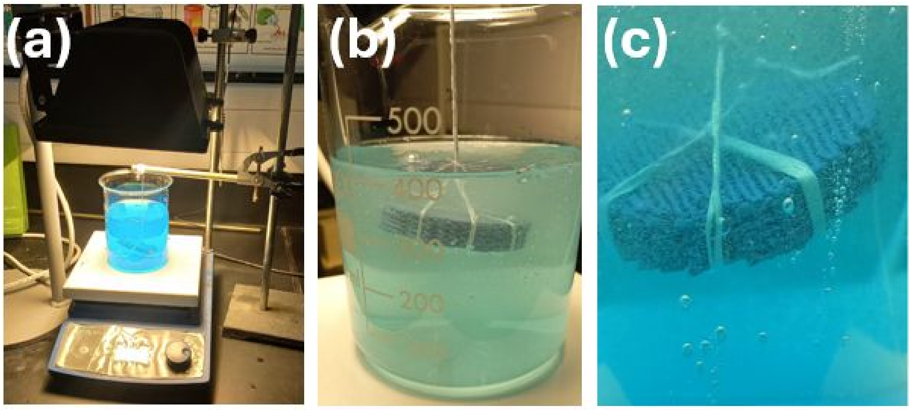

Figure S1: Experimental set up for photocatalysts testing for MB degradation, (a) experimental set up for catalyst testing, (b) picture during the degradation process a clear color change of solution is visible, and (c) zoomed representative image showing evolution of gases during the degradation process.

2. Photocatalytic degradation of MB

The percentage of conversion and/or degradation of MB, was calculated by using the equation presented below:

$$\text{Degradation Rate }(\%) = \frac{(C_0 - C)}{C_0} * 100\% \qquad 1$$

Where C_0 is the initial concentration of MB dye and C is the concentration of dye after irradiation and taken at specific time intervals.

3. N_2-Adsorption and desorption analysis

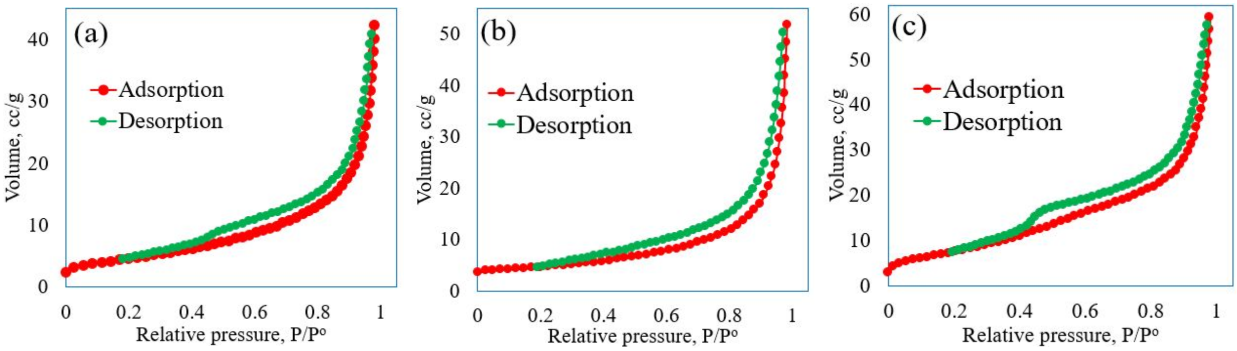

Figure S2: N_2-Adsorption and desorption isotherms of the catalysts after calcination at 600°C, (a) clay, (b) Al_2O_3/Clay, and (c) ZnO/clay alumina.
